# Clarification of anomalies in the application of a 2La molecular karyotyping method for the malaria vector *Anopheles gambiae*

**DOI:** 10.1186/1756-3305-1-45

**Published:** 2008-12-17

**Authors:** Kija R Ng'habi, Claudio R Meneses, Anthony J Cornel, Michel A Slotman, Bart GJ Knols, Heather M Ferguson, Gregory C Lanzaro

**Affiliations:** 1 Ifakara Health Institute, Mlabani Passage, Ifakara, Tanzania; 2 Center for Vectorborne Diseases, University of California, CA, USA; 3 Department of Entomology Texas A&M University College Station, TX, USA; 4 Wageningen University and Research Centre, Wageningen, The Netherlands; 5 Division of Immunity & Infection, and Division of Environmental & Evolutionary Biology, University of Glasgow, Glasgow, UK

## Abstract

**Background:**

Chromosomal inversions have been considered to be potentially important barriers to gene flow in many groups of animals through their effect on recombination suppression in heterokaryotypic individuals. Inversions can also enhance local adaptation in different groups of organisms and may often represent species-specific differences among closely related taxa. We conducted a study to characterize the 2La inversion karyotypes of *An. gambiae sensu stricto *mosquitoes sampled from the Kilombero Valley (Tanzania) using a newly designed PCR assay.

**Results:**

We frequently encountered a (687 bp) fragment which was only present in the Kilombero Valley populations. Laboratory crossing between *An. gambiae s.s. *from Njage (Tanzania) and Kisumu (Western Kenya) populations resulted in F_1 _offspring carrying the observed fragment. Karyotype analysis did not indicate differences in 2La region chromosome morphology between individuals carrying the PCR fragments, the 207 bp fragment, or the 687 bp fragement.

**Conclusion:**

The observed insertion/deletion polymorphism within the region amplified by the 2La PCR diagnostic test may confound the interpretation of this assay and should be well considered in order to maintain an acceptable level of reliability in studies using this assay to describe the distribution and frequency of the 2La inversion among natural populations of *An. gambiae s.s.*

## Background

The *Anopheles gambiae *complex consists of seven closely related species, including two of the most important vectors of malaria in Africa, *An. gambiae s. s. *Giles and *An. arabiensis *Patton. Chromosomal rearrangements in the form of paracentric inversions are common in these species and have been studied extensively in this complex [1-4]. Various 2La chromosome inversions play a role in the subdivision of *An. gambiae s.s. *populations from West and Central Africa [1,2,4,5] and the seven recognised species within the complex can be distinguished by fixed chromosomal arrangements [2,3]. *Anopheles arabiensis *and *An. merus *Dönitz are monomorphic for the 2La arrangement, whereas *An. bwambae *White, *An. melas *Giles and *An. quadriannulatus *Theobald (A & B) are fixed for the alternative arrangement 2L^+a^. *An. gambiae s.s. *is the only complex member in which the 2La inversion is polymorphic having 2L^+a^, 2L^a^/L^+a ^and 2L^a ^arrangements [2,3]. In *An. gambiae s.s.*, it is believed that this chromosome inversion provides adaptation to arid conditions [3,6]. Specifically the spatial distribution of the mosquitoes with different types of 2La inversions is strongly associated with particular habitats. For example the wild type phenotypes (2L^+a^) are associated with wetter climate while the inverted phenotypes (2L^a^) are common in dry climates [7], and its frequency within a population changes in response to seasonal fluctuations in rainfall [1,5,8]. Similarly, the 2La inversions have been linked to microclimatic differences that impact mosquito feeding and resting behaviour. For example, 2La inversion phenotypes have commonly been found resting indoors where there is reduced humidity saturation [8]. Such behavioural heterogeneity may have serious epidemiological impact and may influence the outcome of malaria vector control programmes. For example, the indoor residual spraying (IRS) strategy will not uniformly impact the *An. gambiae s.s. *population [9] as this approach will miss the subpopulation that rests outdoors.

The 2La chromosomal inversion polymorphism is also associated with susceptibility to *Plasmodium *in some 2La phenotypes [10,11], and LRIM1, a major anti-*Plasmodium *gene located within the breakpoints of the 2La arrangement [12], has been shown to be variable for adaptive alleles in *An. gambiae *[13]. Since the 2La inversion is associated with important phenotypes in *An. gambiae*, its frequency and distribution in natural populations is of major interest.

**Figure 1:**
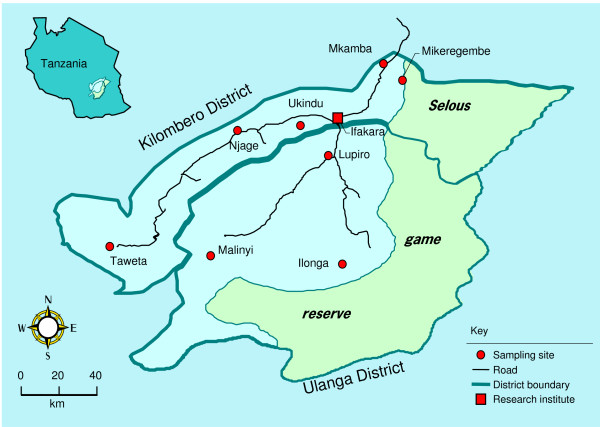
Map of Kilombero/Ulanga District, Tanzania showing mosquito collection sites and origin (Njage) of the laboratory colony *An. gambiae s.s. *used in this study.

A PCR-based assay to determine 2La karyotypes has been developed and extensively validated under field conditions [14,15]. The PCR utilises three primers that produce two products that distinguish 2La karyotypes. A 207 bp fragment is produced if the mosquito carries a standard (un-inverted) arrangement, a 492 bp fragment if it carries the 2La inversion and in heterozygotes the PCR results in two fragments (207/492). In this study we attempted to characterize 2La inversion karyotypes in *An. gambiae s.s*. populations from the Kilombero Valley of Tanzania, whose population genetic structure has not been previously described. Malaria transmission within this area occurs at some of the highest intensities ever described [16,17], and there is thus strong motivation to increase knowledge of the demography, ecology and genetic structure of resident vector populations in order to generate biological insights that could strengthen current and future control strategies. In the course of these studies we observed a PCR band of 687 bp (which was reported by Obbard et al [18]) from individuals collected from Kilombero valley (Fig. 1). To examine the origin and diagnostic relevance of this fragment we performed polytene chromosome analyses, crossing experiments and sequence analysis.

## Materials and methods

### Mosquito collection

Mosquitoes were collected from seven localities within the Kilombero Valley (Tanzania), from January – May 2007: Ilonga, Lupiro, Malinyi, Mikeregembe, Mkamba, Taweta and Ukindu (Fig. 1). In each village, CDC light traps were set in different houses for three consecutive nights. Every morning, traps were retrieved and mosquitoes identified morphologically. Mosquitoes identified as *An. gambiae s.l. *were individually preserved in tubes with silica gel for species-diagnostic PCR [19].

We also analysed 30 male and 79 female *An. gambiae s.s *from a colony (Njage strain) originally established from females collected in Njage village [20,21] about 70 kms away from the Ifakara research Institute (Fig. 1). In addition, we analyzed thirty *An. gambiae s.s. *samples from each of the following colonies maintained at Davis, CA: Kisumu (Western Kenya), Banambani (Mali) and Loum (Cameroon).

### Crossing experiments

Crosses were conducted by placing 100 virgin males with 100 virgin females of the Kisumu and Njage strains together in mating cages. Crosses were done *en masse *(100 females + 100 males), rather than single pair matings. Crosses were conducted in both directions (i.e. Cross 1: Kisumu females × Njage males, Cross 2: Njage females × Kisumu males). The offspring obtained from the two crosses were reared in separate trays to the adult stage. Larvae were fed with fish food Tetramin^® ^and maintained at 27°C. Forty eight male and female F_1 _offspring from each cross were taken for molecular analysis of the 2La inversion karyotype and visual karyotyping by microscopy.

### Molecular methods

Individual mosquitoes were ground in a TissueLyser^® ^for high-throughput disruption of biological samples. DNA purification was carried out using a Qiagen BioSprint 96 workstation following the protocol for DNA extraction from animal tissues as supplied by the manufacturer. Molecular species diagnostic of field-collected mosquitoes was performed as described by Fanello *et al. *[22]. Samples from laboratory colonies (F_0_) and F_1 _progeny of the crosses were subjected to PCR procedures as described by Favia *et al. *[23] to determine molecular forms. All specimens were also screened using the PCR assay for the 2La inversion [15]. This PCR produces a 207 bp fragment for the 2La^+ ^arrangement and a 492 bp fragment for the 2La arrangement. PCR products were visualized in 1.5% agarose gels.

Several 687 bp fragments resulting from the 2La diagnostic PCR of Njage strain samples were purified using a Qiagen PCR purification kit. Purified PCR products were cloned using the TOPO-TA cloning kit (Invitrogen). Plasmid DNA of colonies containing a 687 bp insert was purified using a Mini-prep kit from Qiagen. Inserts were sequenced in both directions on an ABI 3100 Genetic Analyzer, using the M13 forward and reverse primer and ABI Big-Dye Terminator v3.1. Sequences were edited and aligned in the DNA-STAR^© ^package from Lasergene. Sequences were compared to those deposited in the National Center for Biological Information (NCBI). All sequences were submitted to Genbank. Accession Numbers are provided in Table 1.

**Table 1:** Sequence and GenBank accession numbers for 687 bp and 207 bp PCR products generated by the White et al. (2008)

Sequence name	GenBank accession No.	Sequence name	GenBank accession No.
687 bp fragments		207 bp fragment	
Ifakara1f	EU805810	Ifakara19a	EU805818
Ifakara2j	EU805811	Ifakara19b	EU805819
Ifakara8k	EU805812	Ifakara19m	EU805820
Ifakara45a	EU805813	Ifakara60a	EU805821
Ifakara60b	EU805814	Ifakara60e	EU805822
Ifakara78b	EU805815	Ifakara74a	EU805823
Ifakara79a	EU805816	Ifakara77d	EU805824
Lupiro1a	EU805817	Lupiro1e	EU805825

Sequences from *An. gambiae *colonies established from mosquitoes collected from Njage village (Ifakara) and from field collected mosquitoes collected in the village of Lupiro, Kilombero Valley, Tanzania

*An. gambiae *2La molecular karyotype diagnostic.

### Polytene chromosome analysis

This analysis was done for colony mosquitoes only. Twenty nine hours following blood feeding, 79 female mosquitoes from Njage strain had their ovaries removed and preserved in Carnoy's solution for karyotyping following procedures described by Della Torre *et al. *[24]. The same procedure was performed on thirty mosquitoes from the F_0 _and F_1 _progeny of the two populations crossed in this study.

### Ethical approval

The protocol was granted ethical approval by the ethical committee of the Ifakara Health Institute (ref no IHDRC/EC4/CL.N96/2004) and the Tanzanian National Institute of Medical Research (ref no.NIMR/HQ/R.8a/Vol.IX/345).

## Results

A total of 603 field-collected *An. gambiae s.l. *mosquitoes were analysed in this study. Of these, 113 (18.7%) were *An. gambiae s.s. *and 490 (81.2%) were *An. arabiensis. *As expected, all *An. gambiae s.s. *belonged to the S molecular form (the M molecular form has never been found in East Africa [1,2]). Both *An. gambiae s.s*. and *An. arabiensis *were found in all villages except Ukindu (only *An. arabiensis*). However, the relative frequency of the two species varied substantially between villages (Table 2).

**Table 2:** Locations of collection sites and origin of laboratory colonies of *An. gambiae s.l. *mosquitoes used in this study.

				***N***			**2L arrangements and 2L PCR genotypes**					
											
**Country**	**Locality**	**Coordinates (Lat; Long)**	**Source**	*Ag*	*Aa*	**Molecular form**	2L^+a^/2L^+a^		2La/2L^+a^		2La/2La	HWE: χ^2^; P values
							207/207	207/687	207/492	687/492	492/492	
Cameroon	Loum	-4.100; 11.500	Colony	30	0	M	0	0	16	0	14	*P *= 0.6*
Mali	Banambani	-8.050; 12.800	Colony	30	0	M	1	0	12	0	17	*P *= 0.9*
Kenya	Kisumu	-0.583; 34.466	Colony	37	0	S	6	0	22	0	9	*P *= 0.9*
Tanzania	Njage	-8.133; 36.683	Colony	79	0	S	2	3	23	37	14	*P *< 0.001**
Tanzania	Lupiro	-8.377 36.667	Field	45	101	S	10	1	7	8	19	*P *< 0.001**
Tanzania	Mkamba	-8.033; 37.767	Field	51	23	S	18	0	1	2	30	*P *< 0.001**
Tanzania	Mikeregembe	-8.036; 37.967	Field	1	138	S	1	0	0	0	0	-
Tanzania	Ukindu	-8.277; 36.667	Field	0	76	N/A	0	0	0	0	0	-
Tanzania	Malinyi	-8.933; 36.133	Field	1	122	S	1	0	0	0	0	-
Tanzania	Ilonga	-9.067; 36.855	Field	15	15	S	3	0	3	2	7	*P *= 0.5*

Total number of *An. gambiae s.s. (Ag) and An. arabiensis (Aa.) *collected from each locality; molecular form and chromosome 2La arrangement, as determined using the PCR diagnostic of White et al., (2007) are provided. Chi-square *P *= values for observed vs expected, HWE for 2La karyotype frequencies are provided in the far right column.

**Figure 2:**
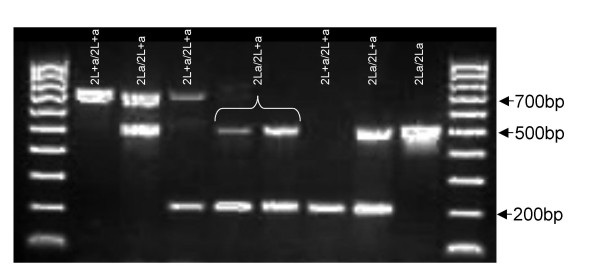
Observed PCR banding patterns from both field and colony *Anopheles gambiae s.s. *mosquitoes from Tanzania and their corresponding polytene chromosome arrangements as visualized using microscopy (the label of each lane stands for the polytene chromosomal arrangement).

**Figure 3:**
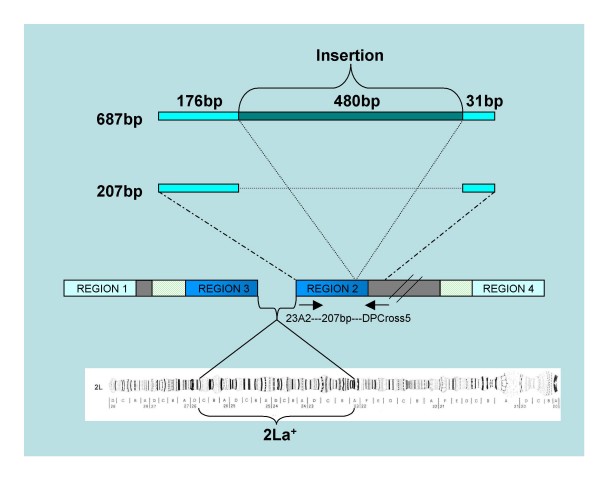
Illustration of the PCR diagnostic for the 2La^+ ^gene arrangement showing the position of the 207 bp fragment produced by the 23A2 and DPCross5 primers and 480 bp insertion we commonly observed in mosquitoes from Tanzania. Modified after White *et al*. (2007).

We subjected each *An. gambiae s.s. *specimen to the 2La molecular karyotyping assay of White *et al. *[15]. We observed the 687 bp fragment from the 2La PCR in 12.4% of field-collected specimens (Table 2, Fig. 2). This fragment (Figure 3) was not present in the Loum (Cameroon), Banambani (Mali) or Kisumu (Kenya) colonies. However, in the Njage colony (Tanzania), it was found in 61.6% of mosquitoes heterozygous for the 2La arrangement (2L^a/^2L^+a^) and 60% of mosquitoes homozygous for the standard arrangement (2L^+a^/2L^+a^), based on polytene chromosome analysis (Table 2). Additionally, specimens that were homozygous for the 687 bp, or heterozygous for the 687 bp and 207 bp fragment were homozygous for the 2La standard arrangement, 2L^+a^/2L^+a ^(Fig. 2, Table 2). Specimens that had both the 687 bp and 492 bp fragments were heterozygotes, 2L^a^/2L^+a ^(Fig. 2, Table 2). The 687 bp fragment was not observed in mosquitoes homozygous for the inverted arrangement, 2L^a^/2L^a ^(Fig. 2, Table 2). No single individual out of the 79 mosquitoes from the Njage colony was found to have all three PCR fragments together, indicating that the 687 bp fragment segregates with 2L^+a^. With the exception of the collection from the village of Ilonga, all populations collected from Kilombero valley showed significant departure from Hardy-Weinberg equilibrium (Table 2).

### Crossing experiments

To examine whether the 687 bp fragment followed a Mendelian pattern of inheritance, we crossed the Njage and Kisumu strains (both S forms) in both directions. A total of 192 F_1 _progeny from the two crosses were analyzed. Based on PCR analysis, the frequencies of chromosome 2La arrangements in the progeny F_1 _mosquitoes are presented in Table 3. The 687 bp gene fragment was present in at least some offspring possessing karyotypes in which it would be expected (2L^+a^/2L^+a ^and 2L^a^/2L^+a^). We observed no 687/687 homozygotes as expected, since this "allele" is absent from the Kisumu colony. Differences in the observed frequencies between crosses are mostly likely the results of sampling errors, since crosses were conducted *en masse*. The results of the crossing experiments further confirm that the 687 bp fragment is not a PCR artifact.

**Table 3:** Distribution of chromosome 2 left arm arrangements in F1 progeny from crosses of the Kisumu and Njage strains in the laboratory.

**Cross**	**2L^+a^/2L^+a^** **207/207**	**2L^+a^/2L^+a^** **207/207**	**2L^+a^/2L^+a^** **207/687**	**2L^+a^/2L^+a^** **207/687**	**2L^+a^/2L^+a^** **687/687**	**2L^+a^/2L^+a^** **687/687**	**2La/2L^+a^** **207/492**	**2La/2L^+a^** **207/492**	**2La/2L^+a^** **687/492**	**2La/2L^+a^** **687/492**	**2La/2La** **492/492**	**2La/2La** **492/492**	**TOTAL**
	F	M	F	M	F	M	F	M	F	M	F	M	
Njage × Kisumu*	5	7	5	0	0	0	15	21	5	2	18	17	95*
Kisumu × Njage	6	7	14	9	0	0	28	27	0	2	0	3	96

*A total of 96 mosquitoes were sampled in both directions. One mosquito DNA did not amplify.

'M' and 'F' stands for male and female respectively.

### Sequence analysis

Clones containing the 687 bp fragment from eight (laboratory and field) individuals and clones with the 207 bp fragment from eight (field and laboratory) individuals were sequenced. GenBank accession numbers are provided in Table 1. A total of thirteen polymorphic sites were observed in both groups, two in the 207 bp group and 11 in the 687 bp group. Sequence analyses showed that the 687 bp fragment is comprised of the 207 bp fragment containing an insertion of 480 bp (Fig. 3). A blast search of this 480 bp insertion against the *An. gambiae *genome [25] indicates that it contains three sequences that are present at least 70–80 times in the genome. That is, the insertion is comprised of repetitive DNA sequences. A comparison of the 687 bp fragment to sequences submitted to GenBank showed that one of the 687 bp fragments we sequenced, showed 100% similarity with a putative 2La chromosomal inversion-assay fragment (Mbt8_2L^+^) observed by Obbard *et al. *[18] using the diagnostic 2La PCR from White *et al *[15] in samples from Mbita (Kenya). Anomalous bands of four different sizes in samples from Western Kenya and Mount Cameroon were reported by Obbard et al., as well as one reported by Slotman et al. [13] in a Cameroonian sample. The Kilombero fragment, however, was similar to one of the fragments found in Western Kenya (Mbita). Furthermore, the sharing of insertion sequence between Mbita and Kilombero may suggest that this phenomenon is not localised, thus further studies are needed to envisage this phenomenon.

## Discussion and conclusion

The 2La inversion in *An. gambiae *is linked to phenotypes that are important to malaria transmission. These include drought tolerance, endophily [11] and susceptibility to *Plasmodium *infection [12,13,26]. Studies aimed at describing the distribution of the 2La inversion among natural populations are therefore of great interest. The PCR-based method developed by White et al. [15] to facilitate the determination of the 2La karyotype is a significant contribution to *An. gambiae *population biology. The method developed by White *et al*. [15] utilises PCR primers based on sequences around the 2La breakpoints provided by Sharakhov *et al. *[14]. The application of this assay is confounded by polymorphism in the region amplified by this PCR-based method, resulting in the production of fragments inconsistent with those described in the original paper [13,18]. We encountered such a polymorphism that produced an "atypical' fragment of 687 bp which occurred at varying frequencies in *An. gambiae s. s. *populations at several sites in the Kilombero Valley of Tanzania Table 2.

The occurrence of an apparently high degree of insertion/deletion polymorphism within the sequence amplified by the White *et al. *PCR diagnostic [15] may confound its application. We confirm that this fragment can be reliably detected through the robust 2La inversion karyotyping techniques previously developed for *An. gambiae s.s. *mosquitoes [15]. Our results indicate that the 678 bp fragment is not an artifact, but a result of insertion/deletion polymorphism within the region amplified by the 2La PCR diagnostic. Therefore, the amplification of these unexpected fragments may confound the interpretation of this assay and we recommend that they should be well considered in order to maintain an acceptable level of reliability in studies aimed at describing the distribution and frequency of the 2La inversion among natural populations of *An. gambiae s.s.*

## Competing interests

The authors declare that they have no competing interests.

## Authors' contributions

KRN, CRM, GCL designed the experiment of this protocol. KRN, CRM, AC, carried out the laboratory work. KRN, MS, CRM and GCL analyzed and interpreted the data. KRN, CRM, HMF, BGJK drafted the manuscript. All authors read and approved the manuscript.
